# Whole transcriptomic analysis reveals overexpression of salivary gland and cuticular proteins genes in insecticide-resistant *Anopheles arabiensis* from Western Kenya

**DOI:** 10.1186/s12864-024-10182-9

**Published:** 2024-03-27

**Authors:** Diana Omoke, Lucy Mackenzie Impoinvil, Dieunel Derilus, Stephen Okeyo, Helga Saizonou, Nicola Mulder, Nsa Dada, Audrey Lenhart, Luc Djogbénou, Eric Ochomo

**Affiliations:** 1 Kenya Medical Research Institute (KEMRI), Centre for Global Health Research (CGHR), Kisumu, Kenya; 2 Entomology Branch, Division of Parasitic Diseases and Malaria, Centers for Disease Control and Prevention, Atlanta, USA; 3 University of Abomey Calavi (UAC), Abomey Calavi, Benin; 4 University of Cape Town ZA, Cape Town, South Africa; 5 School of Life Sciences, Arizona State University, Tempe, AZ USA; 6 Tropical Infectious Disease Research Center, University of Abomey- Calavi, Abomey Calavi, Benin

**Keywords:** Insecticide resistance, Malaria, *Anopheles arabiensis*, RNA-Seq, Whole transcriptomic sequencing

## Abstract

**Background:**

Effective vector control is key to malaria prevention. However, this is now compromised by increased insecticide resistance due to continued reliance on insecticide-based control interventions. In Kenya, we have observed heterogenous resistance to pyrethroids and organophosphates in *Anopheles arabiensis* which is one of the most widespread malaria vectors in the country. We investigated the gene expression profiles of insecticide resistant *An. arabiensis* populations from Migori and Siaya counties in Western Kenya using RNA-Sequencing. Centers for Disease Control and Prevention (CDC) bottle assays were conducted using deltamethrin (DELTA), alphacypermethrin (ACYP) and pirimiphos-methyl (PMM) to determine the resistance status in both sites.

**Results:**

Mosquitoes from Migori had average mortalities of 91%, 92% and 58% while those from Siaya had 85%, 86%, and 30% when exposed to DELTA, ACYP and PMM, respectively. RNA-Seq analysis was done on pools of mosquitoes which survived exposure (‘resistant’), mosquitoes that were not exposed, and the insecticide-susceptible *An. arabiensis* Dongola strain. Gene expression profiles of resistant mosquitoes from both Migori and Siaya showed an overexpression mainly of salivary gland proteins belonging to both the short and long form D7 genes, and cuticular proteins (including CPR9, CPR10, CPR15, CPR16). Additionally, the overexpression of detoxification genes including cytochrome P450s (CYP9M1, CYP325H1, CYP4C27, CYP9L1 and CYP307A1), 2 carboxylesterases and a glutathione-S-transferase (GSTE4) were also shared between DELTA, ACYP, and PMM survivors, pointing to potential contribution to cross resistance to both pyrethroid and organophosphate insecticides.

**Conclusion:**

This study provides novel insights into the molecular basis of insecticide resistance in *An. arabiensis* in Western Kenya and suggests that salivary gland proteins and cuticular proteins are associated with resistance to multiple classes of insecticides.

**Supplementary Information:**

The online version contains supplementary material available at 10.1186/s12864-024-10182-9.

## Background

The main malaria vector control methods in Kenya include the use of insecticide treated nets (ITNs) containing pyrethroids and indoor residual spraying (IRS) using organophosphates and neonicotinoids [1, 2]. The continued use of these insecticide-based interventions has led to increased resistance among malaria vectors in Kenya, where resistance to all four traditional classes of public health insecticides (pyrethroids, organophosphates, organochlorines and carbamates) have been reported [3]. Recently, resistance to neonicotinoids – a new class of insecticide used in IRS in many countries in sub-Saharan Africa (SSA) – was reported with clothianidin in Central Africa, raising an alarm and highlighting the urgent need for close and timely monitoring of vector susceptibility [4].

*Anopheles arabiensis* is one of the principal vectors of malaria in Kenya, as well as *An. gambiae s.s* and *An. funestus* [5, 6] and recently, *An. stephensi* has been detected in Northern Kenya [7]. Behavioral plasticity of *An. arabiensis* has been shown to compromise the protective effects of ITNs, as they bite both indoors and outdoors throughout the night [8]. Several studies conducted in western Kenya have reported insecticide resistance in *An. arabiensis* as well as other malaria vector species, causing great concern to the National Malaria Control Program (NMCP) [6, 9–12].

Metabolic resistance and target site mutations are the most widely studied and reported mechanisms of resistance. Metabolic resistance involves large enzyme families including cytochrome P450s (CYP450s), carboxylesterases (COEs) and glutathione-s-transferase (GSTs) which are known to confer resistance in malaria vectors. These enzymes exist naturally in mosquitoes and their amplification or overexpression leads to heightened detoxification of insecticides making the mosquitoes resistant [13]. Target site resistance arises from point mutations that alter the insecticide binding sites or transportation channels inside the mosquito [13]. Other modes behind insecticide resistance include: cuticular modifications such as thickening of the cuticles or change in cuticle composition, which prevent or slow insecticide penetration [14]; and behavioral resistance which results in mosquitoes avoiding surfaces treated with insecticides or in mosquitoes that are not affected by spatial repellents [15]. Investigating gene expression profiles of insecticide resistant malaria vectors is important in understanding the underlying mechanisms and in identification of markers that can be used for monitoring purposes. So far, several studies have identified marker genes associated with both pyrethroid and organophosphate resistance in *Anopheles* mosquitoes due to their high expression levels [16–18]. In an *An. arabiensis* population from Ethiopia, genes including CYP9K1, CYP9L1, GSTE4 as well as COEs were associated with metabolic resistance [17]. In addition to detoxification genes, a study conducted by Isaacs, et al. [18] showed that salivary gland proteins were implicated in insecticide resistance.

In this study, we sought to explore insecticide resistance mechanisms using RNA-Seq to identify markers associated with resistance to DELTA, ACYP and PMM in *An. arabiensis* populations from Migori and Siaya counties in western Kenya. These results will inform the NMCP decision making around IRM to ensure the efficacy of vector control interventions.

## Results

### Phenotypic insecticide resistance of *An. arabiensis* from Migori and Siaya

**Fig. 1:**
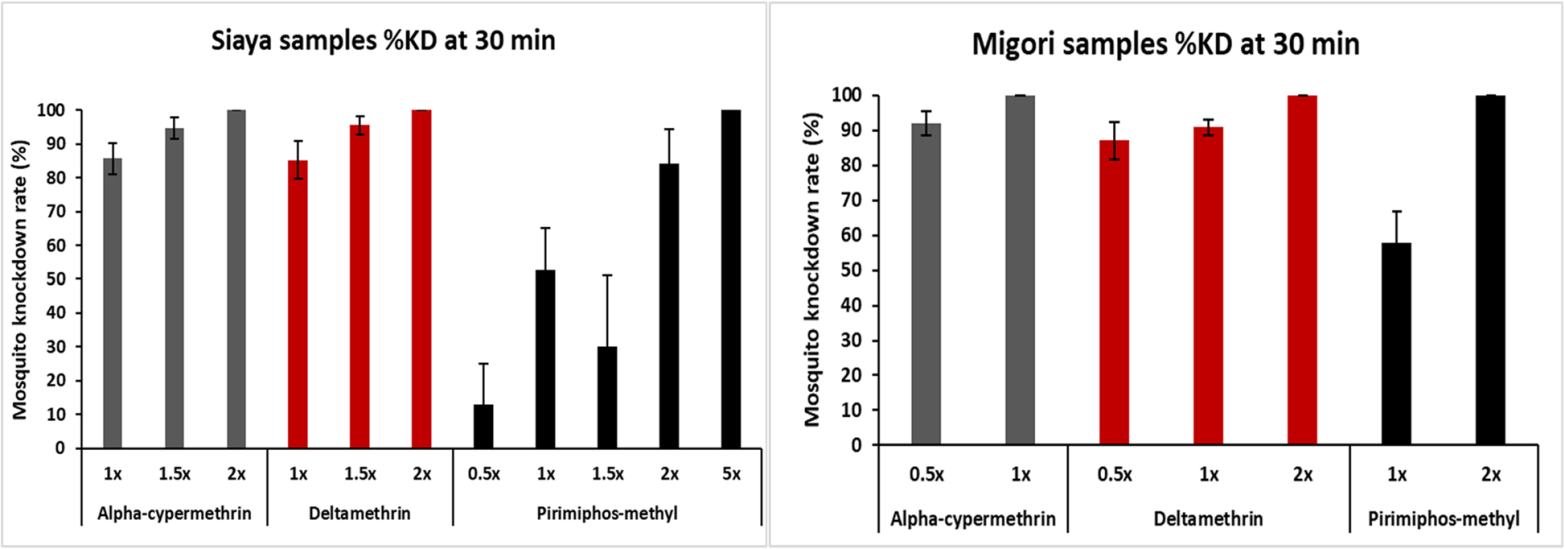
Determination of phenotypic insecticide resistance profiles of *An. arabiensis* from Siaya and Migori counties using CDC bottle bioassays. The average mortalities of mosquitoes after 30 min of insecticide exposure are shown as percentages on the y-axis with 95% confidence intervals

A total of 2404 and 2424 mosquitoes from Migori and Siaya counties, respectively, were exposed to either ACYP, DELTA or PMM. Out of those, 120 mosquitoes from each site (30 mosquitoes surviving exposure to each insecticide and 30 that were not exposed) were pooled in groups of 10 and sequenced alongside three pools of 10 susceptible *An. arabiensis* from the Dongola reference strain. In Migori, 100% mortality was observed at the diagnostic dose (1X) of ACYP, 2X DELTA and 2X PMM, while in Siaya, complete mortality was observed at 2X ACYP, 2X DELTA and 5X PMM. The Migori samples had an average mortality of 91% to 1X DELTA, 92% to 0.5X ACYP and 58% to 1X PMM. In Siaya, the average mortality was at 85% to 1X DELTA, 86% to 1X ACYP and 30% to 1.5X PMM. PCR tests conducted on the legs of the bioassayed mosquitoes confirmed that they were all *An. arabiensis* (Fig. 1).

### Descriptive summary statistics of RNA-Seq data

Whole transcriptomic analysis was done on mosquitoes resistant to DELTA, ACYP, and PMM, the unexposed mosquitoes from both Migori and Siaya as well as the susceptible Dongola mosquito strain. The total number of paired-end reads generated for all the samples were 2.18 billion ranging from 26 – 107 million reads per library. Samples collected from Migori had a total of 1 billion reads ranging from 56 – 107 million. Siaya had 921 million reads ranging from 26 – 101 million while the susceptible Dongola *An. arabiensis* generated a total of 228 million reads ranging from 69 – 85 million. From all the samples, in average 98% of the reads were retained after filtering and removal of adapters and in average 52% of the reads were mapped to the *An. arabiensis* Dongola reference genome (Additional file 1). A typical low mapping rate of RNA-Seq against the reference genome is not surprising and can be attributed to two factors: inherent incomplete ribosomal depletion and the presence of large number of multi-mapped reads that are not reported here due to their meaningless in DEG analysis. The feature counts results showing the number of tags generated and the percentages assigned to exons in the sense orientation has been summarized in Additional file 2 and Additional file 3. The percentage of tags assigned to exons in the sense direction ranged from 43 to 64% (Additional file 2, Additional file 3).

### Differential gene expression associated with alphacypermethrin resistance

Three pairwise comparisons were done to determine the genes which were differentially expressed for ACYP in both Migori and Siaya (Table 1). For Migori, a total of 1088 (795 up and 293 down regulated), 1101 (630 up and 471 down regulated), and 317 genes (160 up and 157 down regulated) were significantly differentially expressed (FDR ≤ 0.01, |FC|≥ 2) in Res-Sus (MA *vs* DO), Con-Sus (MU *vs* DO) and Res-Con (MA *vs* SU), respectively (Table 1, Additional file 4A). For Siaya, a total of 1225 (838 up and 387 down regulated), 1155 (815 up and 340 down regulated) and 63 genes (26 up and 37 down regulated) were significantly differentially expressed in the Res-Sus (SA *vs* DO), Con-Sus (SU *vs* DO) and Res-Con (SA *vs* SU), respectively (Table 1, Additional file 4B).

**Table 1:** Results showing summaries of differential gene expression patterns of *An. arabiensis* for alphacypermethrin, deltamethrin and pirimiphos-methyl. Genes that were significantly differentially expressed at p < 0.01 and fold change (FC) > 2 were considered as candidate genes of interest

Sample ID	No. of genes tested	DE genes (adjP < 0.01)		DE genes(|FC|> 2 adjP < 0.01)	
		**Up**	**Down**	**Up**	**Down**
**SA vs SU**	9984	42	45	26	37
**SD vs SU**	10,006	34	42	26	34
**SP vs SU**	10,217	84	43	42	26
**SA vs DO**	10,217	1501	1202	838	387
**SD vs DO**	10,231	1764	1118	856	331
**SP vs DO**	10,386	2096	1923	987	509
**SU vs DO**	10,265	1089	617	815	340
**MA vs MU**	9707	933	764	160	157
**MD vs MU**	9626	204	296	117	201
**MP vs MU**	9814	980	1138	193	164
**MA vs DO**	10,217	1495	820	795	293
**MD vs DO**	10,204	833	374	657	249
**MP vs DO**	10,249	1982	1741	1007	500
**MU vs DO**	10,158	1570	1554	630	471

*SA* Siaya alpha-cypermethrin, *SD* Siaya deltamethrin, *SP* Siaya pirimiphos-methyl, *SU* Siaya unexposed, *MA* Migori alpha-cypermethrin, *MD* Migori deltamethrin, *MP* Migori primiphos-methyl, *MU* Migori unexposed, *DO* Dongola (*An. arabiensis* reference susceptible strain), *DE* differentially expressed, *FC* Fold change, *adjP* P-value adjusted for multiple testing by the method of Benjamini and Hochberg 1995

The list of differentially expressed genes (DEGs) that are shared between two or more comparisons (Res-Sus, Con-Sus, Res-Con), as described in Additional file 4, were extracted and mapped to their Fold Change expression and functional description (Additional file 5). Focusing on the significantly differentially expressed genes shared between Res-Sus and Res-Con, Migori had a total of 70 DEGs (66 up and 4 down regulated) (Additional file 4A). Among the top 5 genes with retrievable annotations included a serine protease 53-like, a microfibril-associated glyco 4-like, a synaptic vesicle glyco 2B-like, a senecionine N-oxygenase and an uncharacterized protein. This group also included 2 cuticular proteins (flexible cuticle 12-like and adult cuticle 1-like), a carboxylesterase-6 like, and 3 cytochrome P450s (CYP307A1, CYP4C36 and CYP6M2). Siaya had a total of 23 DEGs (17 up and 6 down regulated) (Additional file 4B). The top 5 up regulated genes with retrievable annotations included 3 cuticular proteins (1 cuticular protein CPLCG family and 2 cuticular protein CPLCG family), an ATP-binding cassette transporter and a basic proline-rich -like gene. No detoxification genes were detected in this group.

It is worth noting that in all Res-Sus ACYP comparisons from both sites, the DEGs associated with cuticular proteins (CPs) and salivary gland proteins (SGPs), were predominantly overexpressed at higher proportions compared to detoxification genes. In addition, CPs, SGs and GSTs had higher ratios of up regulated genes (Additional file 6). Among the annotated DEGs in Migori, 92% of cuticular (34/37), 97% salivary (28/29), 72% CYP450s (13/18), 86% GSTs (6/7), and 50% COEs (3/6) were up regulated in the Res-Sus comparisons. In Siaya, 90% of cuticular (37/41), 96% salivary (25/26), 65% CYP450s (13/20), 90% GSTs (9/10), and 50% COEs (2/4) were up regulated (Additional file 6).

**Fig. 2:**
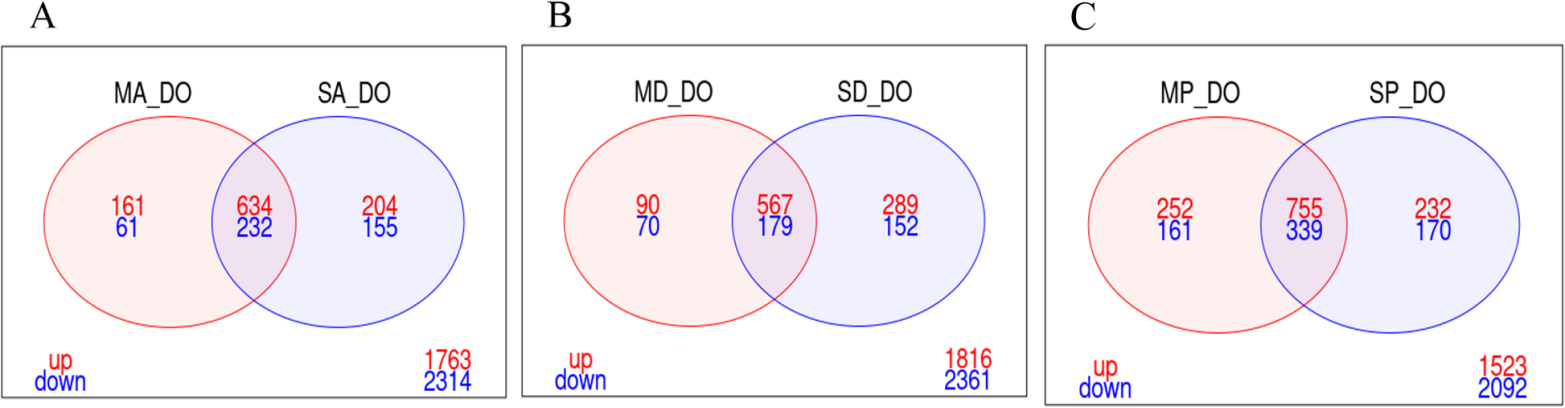
Venn diagram of differentially expressed genes (log2FC > 1 and FDR < 0.01), showing the number genes that commonly differentially expressed in the resistant populations of the two sites for each insecticide. **A** Alpha-cypermethrin; **B** Deltamethrin; and; **C** Pirimiphos-methyl

Identification of core differentially expressed salivary gland proteins, cuticular proteins and the detoxification genes associated with ACYP resistance was performed by selecting genes commonly shared in the Res-Sus comparisons from both sites. Here, there was a total of 30 cuticular proteins (28 up and 2 down regulated) and 25 salivary gland protein genes (24 up and 1 down regulated). The up regulated cuticular protein genes included CPAP3-A1b, CPAP3-Az1c, CPR10, CPR15, CPR16 and CPR9 while salivary gland protein genes included D7L1 and D7L2 (Fig. 2A, Additional file 7). There was a total of 26 detoxification genes which were differentially expressed in the ACYP Res-Sus comparisons across both sites. These included the following 18 (10 cytochromeP450s, 6 GSTE and 2 COEs) up regulated genes: CYP307A1, CYP4C27, CYP4H15, CYP6M2, CYP6M3, CYP6P4, CYP6Z3, CYP9K1, CYP9L1, CYP9M1, GSTD12, GSTD7, GSTE4, GSTE7, GSTE8, GSTU1 & 2 COEs as well as the following 8 (5 cytochromeP450s, 1 GSTE and 2 COEs) down regulated genes: CYP302A1, CYP325D1, CYP325H1, CYP4C35, CYP9M2, GSTE1 and 2 COEs (Table 2).

**Table 2:** Table showing differentially expressed genes in the resistant vs susceptible (Res-Sus) and unexposed vs susceptible pairwise comparisons. FDR =  < 0.05, FC =  > 2

Gene Type	Gene ID	Gene Description	Migori ACYP R-S	Migori DELTA R-S	Migori PMM R-S	Migori UNEXP C-S	Siaya ACYP R-S	Siaya DELTA R-S	Siaya PMM R-S	Siaya UNEXP C-S
**Cuticular proteins**	AARA000220	cuticular protein	3.73	2.69	6.68		4.08	4.08	4.69	5.13
	AARA001131	cuticular protein RR-2 family		8.06	2.07		4.86	2.57		
	AARA001390	cuticular protein	2.01	2.51						
	AARA001419	cuticular protein	0.39			0.36		0.46	0.20	0.22
	AARA001471	cuticular protein RR-2 family							0.15	
	AARA002110	cuticular protein RR-2 family							0.26	
	AARA002342	CPR16	7.41	9.25	11.79	6.87	10.48	8.75	6.41	9.13
	AARA002344	CPR15	2.83	4.35	4.14	3.32	3.34	3.34	2.22	2.89
	AARA002509	cuticular protein RR-2 family				0.28				
	AARA002622	cuticular protein RR-1 family	2.10	3.73	3.05		2.71	2.68		
	AARA003675	cuticular protein RR-2 family	19.84	12.38	6.06	15.89	14.83	14.93		
	AARA003897	cuticular protein RR-1 family	4.99	8.94	6.50	5.10	7.62	5.78	4.47	4.89
	AARA003899	cuticular protein RR-1 family	5.74	6.50	8.22	4.03	4.86	4.38	4.47	6.15
	AARA003903	cuticular protein RR-1 family	8.75	13.36	12.55	5.39	9.71	11.16	6.92	6.15
	AARA003986	cuticular protein	2.17	2.68	2.85		2.89	2.93	2.10	
	AARA004015	cuticular protein CPLCP12					2.14			
	AARA004816	CPR10	2.31	2.62	3.78		2.91	2.79	3.41	2.31
	AARA005816	cuticular protein RR-2 family			3.27					
	AARA006486	cuticular protein TWDL family							4.35	4.96
	AARA007248	cuticular protein RR-2 family			0.19	0.24			0.21	0.37
	AARA007522	cuticular protein RR-1 family	0.44				0.43		0.24	0.42
	AARA008818	cuticular protein RR-2 family								
	AARA009171	CPR59							0.34	0.41
	AARA009194	cuticular protein RR-1 family				0.39				
	AARA011115	cuticular protein CPLCG family	3.41	16.22			9.00	2.71		
	AARA011117	cuticular protein CPLCG family		17.51						
	AARA011120	cuticular protein CPLCG family	4.56	5.62	8.22	4.14	4.99	4.63	4.44	
	AARA011324	cuticular protein 3 from 51 aa family			0.06	0.10		0.17	0.11	0.05
	AARA015614	CPR9	3.51	3.41	3.46	3.89	4.20	3.43	4.41	6.28
	AARA015776	cuticular protein RR-2 family		0.01	0.06	0.07	0.10		0.04	0.09
	AARA016140	cuticular protein RR-1 family	6.06	6.11	15.14	5.35	8.28	6.54	7.67	6.02
	AARA016147	cuticular protein RR-1 family	4.89	4.53	5.28		3.56	3.92		4.69
	AARA016150	cuticular protein RR-1 family	2.66	3.03	2.55	3.81			2.50	2.27
	AARA016552	CPAP3-A1c	2.36	2.81		3.41	2.41	3.05		
	AARA016553	CPAP3-A1b	4.06	3.01	3.97	4.76	3.01	3.63	3.29	3.10
	AARA017512	cuticular protein CPLCG family		7.26	2.57		4.35	3.46		
	AARA017766	cuticular protein RR-2 family								
	AARA000730	chitin deacetylase 1 isoform X2		2.58	2.73	2.58	2.31	2.30	2.04	
	AARA003900	larval cuticle LCP-30-like	4.53	8.94	5.66	3.94	4.50	5.46	4.79	3.61
	AARA005143	cuticle 1				0.32	2.10		0.33	0.34
	AARA005763	chitinase	2.28	3.01	2.11		2.41	2.16		2.23
	AARA005785	pupal cuticle 36-like isoform X1			5.50				2.51	4.82
	AARA005787	pupal cuticle 36-like	5.10	4.92	4.79	2.60	4.92	5.35	5.13	5.28
	AARA005953	chitin-binding domain cbd-1-like			2.46					
	AARA005955	probable chitinase 10		0.25	0.14		0.35	0.20	0.28	
	AARA007063	endochitinase-like				16.22				
	AARA007329	chitinase	99.04	184.82	137.19	67.18	205.07	191.34	294.07	101.83
	AARA007725	cuticle -like	2.71	3.51	2.20		2.14	3.51	3.05	
	AARA008295	chitinase								
	AARA008306	probable chitinase 10			2.79		2.53		2.93	
	AARA008307	chitinase partial			3.20		3.41	2.11	3.53	
	AARA009226	chitinase	2.08	2.57	2.75		2.25	2.75	2.27	
	AARA009427	endochitinase A		0.45		0.49				
	AARA010757	venom allergen	2.62	2.55	2.19		3.23	3.12		
	AARA011020	probable chitinase 10	4.00	4.86	3.73				3.03	3.05
	AARA017101	endocuticle structural glyco ABD-4	0.38	0.26	0.09	0.50	0.12	0.17	0.07	0.05
	AARA017258	probable endochitinase								5.62
	AARA017495	probable chitinase 10	13.55						7.26	18.25
	AARA017497	probable chitinase 10	53.08	57.28	74.03		42.52	57.28	48.17	60.13
	AARA017618	endochitinase-like isoform X2	2.31					2.22	3.18	3.20
	AARA017619	endochitinase-like					3.07		4.59	
	AARA017620	chitinase	3.61	3.03	3.36		3.71	4.92	7.94	5.66
	AARA017621	endochitinase-like	3.61	3.43	3.66	2.30	3.39	4.23	4.99	4.17
	AARA017622	chitinase			2.14	2.03	2.01			
	AARA017810	chitin-binding domain cbd-1-like	3.66		5.06	2.51	3.05	3.18	3.86	2.97
	AARA017994	chitinase	79.34						46.21	
**Salivary Gland proteins**	AARA001015	salivary 15		3.18	2.35	2.46	2.01			
	AARA001016	salivary 15		2.83	2.03					
	AARA007686	kDa salivary	7.21	6.19	10.63	10.20	6.59	6.45	7.73	7.73
	AARA008319	probable salivary secreted peptide	0.49			0.33	0.40	0.44	0.43	
	AARA008506	salivary gland protein	2.62	2.38	2.97		2.57	2.45	2.03	2.13
	AARA009912	salivary secreted peptide			2.08					
	AARA010474	kDa salivary	2.69		4.23	2.64				2.95
	AARA011261	salivary hyp10	3.73	2.38	4.06	3.86	2.43	2.46	2.60	3.10
	AARA011262	salivary 12	4.76	3.23	5.82	5.28	3.10	3.29	4.14	4.03
	AARA015881	salivary secreted peptide	2.75		2.14		2.19	2.58	2.25	2.13
	AARA016176	sg2a salivary	18.00	9.58	12.82	19.16	9.85	14.72	11.16	8.11
	AARA016215	probable salivary secreted peptide			3.12				2.38	3.61
	AARA017300	secreted salivary gland		0.41	0.33	0.10			0.35	0.18
	AARA017669	kDa salivary secreted	5.06	4.06	7.36	2.01	5.98	4.35	6.68	7.01
	AARA018091	kDa salivary	5.24	6.82	5.94	3.43	4.29	8.34	8.94	9.32
	AARA001829	TRIO salivary gland protein	3.29	3.66	3.05	2.43	3.01	2.48	2.41	2.01
	AARA008387	probable salivary secreted peptide	6.87	4.08	6.06	6.59	3.51	3.97	4.69	4.20
	AARA009227	basic tail-containing salivary secreted peptide	2.38	2.85						
	AARA009957	salivary gland protein	4.96						2.41	5.13
	AARA010442	salivary gland protein 1-like	3.36		3.78	2.81	3.94	3.53	2.91	2.28
	AARA011280	D7 long form salivary protein			2.22	2.91				
	AARA014717	salivary gland protein	8.57	5.28	7.16	9.45	6.06	6.41	6.32	6.87
	AARA015664	14.5 kDa salivary peptide	2.08				2.03	2.14	2.30	
	AARA016088	salivary gland protein 7-like	3.53	4.14	3.27	2.50	2.68	2.50	2.53	2.60
	AARA016089	salivary gland protein		2.45						
	AARA016177	salivary gland protein 2-like	4.76	4.72	4.08	4.44	3.05	3.36	3.41	
	AARA016193	SG8			2.03					
	AARA016208	salivary gland protein	2.57	2.16	2.58	2.53			2.68	2.69
	AARA016220	salivary gland protein	2.16	2.85	2.66		2.07		2.06	2.19
	AARA016221	salivary gland protein 1-like	2.95	3.56	3.94	3.48	2.83	2.91	2.95	2.53
	AARA016222	salivary gland protein 1-like	2.07	2.33	2.46		2.03		2.00	2.23
	AARA016223	salivary gland protein 1-like	3.12	3.58	4.11	3.03	3.14	2.68	3.25	2.36
	AARA016236	D7 short form salivary protein	4.06	6.77	3.81	4.47	4.00	3.18	3.12	2.79
	AARA016237	D7 short form salivary protein	30.70	46.21	40.50	39.12	31.78	27.28	33.13	26.72
	AARA016239	D7 short form salivary protein	2.77	3.66	2.99	2.97	2.51	2.46	2.35	
	AARA016240	D7 short form salivary protein		2.31	2.01					
	AARA016540	D7L2	2.51	3.66	2.91	2.03	2.64	2.36	2.06	
	AARA016541	D7L1	2.79	4.86	3.16	2.77	3.36	2.75	2.23	
**Cytochrome P450s monooxygenases**	AARA000237	CYP9M1	2.19	2.91	2.62	3.29	2.87	2.68	3.63	
	AARA002507	CYP9K1	2.60		3.53	2.60	3.66	2.95	3.05	3.41
	AARA002563	CYP4G17			2.25					
	AARA003376	CYP9J5								
	AARA003631	CYP325D1	0.26	0.17	0.29	0.46	0.20	0.21	0.19	0.28
	AARA003697	CYP303A1			0.38					
	AARA004308	CYP325K1			4.11				2.97	
	AARA004446	CYP314A1		0.41		0.40				
	AARA004676	CYP6AA1					2.07	2.06	2.07	
	AARA005652	CYP49A1								0.38
	AARA006801	CYP325H1	0.26	0.19	0.25		0.15	0.27	0.16	0.36
	AARA007526	CYP302A1	0.40	0.48		0.30	0.49		0.36	0.41
	AARA008771	CYP4C35	0.28	0.31	0.27	0.33	0.25	0.32	0.30	0.33
	AARA008772	CYP4C36	2.33							
	AARA008776	CYP4C27	2.79	2.69	2.28		2.46	2.53	2.57	
	AARA008798	CYP306A1			0.38	0.33	0.48	0.41	0.37	0.40
	AARA010190	CYP4AA1			2.23	2.13				2.13
	AARA011200	CYP4H17					2.11		2.77	
	AARA011787	CYP4G16	2.04							
	AARA015586	CYP9J4				2.22				
	AARA015607	CYP307A1	3.66	3.23			3.86	3.43	3.78	
	AARA015620	CYP9L		0.41						
	AARA015621	CYP9L1	3.32	2.83	3.29	2.77	3.03	3.32	3.07	3.16
	AARA015635	CYP4H15	2.51		2.14	2.13	2.19			
	AARA015642	CYP6M3	2.75		3.29		3.97	2.95	4.99	4.20
	AARA015644	CYP6M2	4.20		8.34		9.58	5.54	14.93	6.50
	AARA015661	CYP4J10		0.30		0.34		0.35		
	AARA015669	CYP4H19				32.90				
	AARA015787	CYP6P3		0.48		0.40				
	AARA015789	CYP6P4	4.53		5.28		8.51	5.39	5.94	9.00
	AARA015791	CYP6P2	2.38		3.27				2.28	3.16
	AARA015794	CYP6Z1			2.41		2.25	2.06	2.71	2.46
	AARA015795	CYP6Z3	2.20		3.05		3.68	2.60	4.79	2.03
	AARA015844	CYP4H27				2.03				
	AARA015895	CYP9M2	0.24		0.42	0.47	0.41	0.34	0.41	
	AARA016565	CYP4D17				0.37	0.44	0.46	0.38	0.35
	AARA015793	cytochrome P450 6d3-like				0.42			2.46	
**Glutathion-S-transferase**	AARA002574	GSTT2			2.04					2.03
	AARA005891	GSTS1			2.19		2.41	2.11		
	AARA008729	GSTE8	2.50		2.41	2.22	2.17	2.23	2.27	
	AARA008732	GSTE2								0.34
	AARA008734	GSTE7	2.62		3.89	3.43	2.77	3.12	3.05	3.23
	AARA009632	GSTD7	2.45		3.51	2.19	2.97	2.50	3.25	2.79
	AARA011689	GSTU1	2.22			2.17	2.81	2.55		2.03
	AARA015648	GSTE4	3.81	3.16	3.20	2.77	4.26	3.58	3.56	3.01
	AARA015649	GSTE5		2.55	2.36	2.41			2.14	
	AARA015681	GSTMS2					2.06		2.04	
	AARA015682	GSTMS1			2.39		2.22		2.58	2.36
	AARA015729	GSTE1	0.26	0.17	0.20	0.14	0.26	0.21	0.19	0.16
	AARA015763	GSTD12	2.57		2.89	2.31	2.41	2.28	2.73	
	AARA015764	GSTD3							2.06	
	AARA015765	GSTD10			3.23			7.06		
**Carboxylesterase**	AARA001215	carboxylesterase	0.46	0.35				0.44		
	AARA004790	carboxylesterase clade H	3.53	2.99	3.73	4.17	3.94	3.81	3.71	3.29
	AARA016305	carboxylesterase			2.31					
	AARA016468	carboxylesterase	8.69	8.88	12.13	8.88	10.13	11.71	8.51	8.17
	AARA017720	carboxylesterase								
	AARA018213	carboxylesterase				2.04				
	AARA018338	carboxylesterase								
	AARA005879	liver carboxylesterase 4	0.40		0.44	0.44	0.40		0.27	0.41
	AARA016024	Carboxylic ester hydrolase	0.49				0.47	0.41		
	AARA016026	Carboxylic ester hydrolase	2.50	2.16	2.31			2.79		
	AARA017332	Carboxylic ester hydrolase			2.30				3.58	

### Differential gene expression associated with deltamethrin resistance

In Migori, a total of 906 genes (657 up and 249 down regulated) were significantly differentially expressed in mosquitoes resistant to deltamethrin compared to the susceptible strain (MD vs DO). In a pairwise comparison of deltamethrin resistant and the unexposed mosquitoes (MD vs MU), there was a total of 318 differentially expressed genes (117 up and 201 down regulated). A total of 1101 genes (630 up and 471 down regulated) were differentially expressed in the pairwise comparison of the unexposed and susceptible mosquitoes (MU vs DO) (Table 1, Additional file 4A). In Siaya, there was a total of 1187 significantly differentially expressed genes (856 up and 331 down regulated) in the deltamethrin resistant vs. susceptible pairwise comparison (SD vs DO). In the resistant and unexposed pairwise comparison (SD vs SU), there was a total of 60 differentially expressed genes (26 up and 34 down regulated). The pairwise comparison of unexposed and susceptible mosquitoes (SU vs DO) had a total of 1155 differentially expressed genes (815 up and 340 down regulated) (Table 1, Additional file 4B).

Migori had a total of 58 (40 up and 18 down regulated) significantly differentially expressed genes that were shared between Res-Sus and Res-Con (Additional file 4A). Some of the top up regulated genes with retrievable annotations included 3 cuticular proteins, a cytochrome P450 (CYP307A1), a carboxylesterase (AARA001215), probable chitinase and 2 serine proteases. In Siaya, there was a total of 24 differentially expressed genes (17 up and 7 down regulated) shared between Res-Sus and Res-Con (Additional file 4B). The up regulated genes included 4 cuticular proteins: cuticle 7-like, 2 adult cuticle 1-like and histidine-rich PFHRP-II (Additional file 5).

**Fig. 3:**
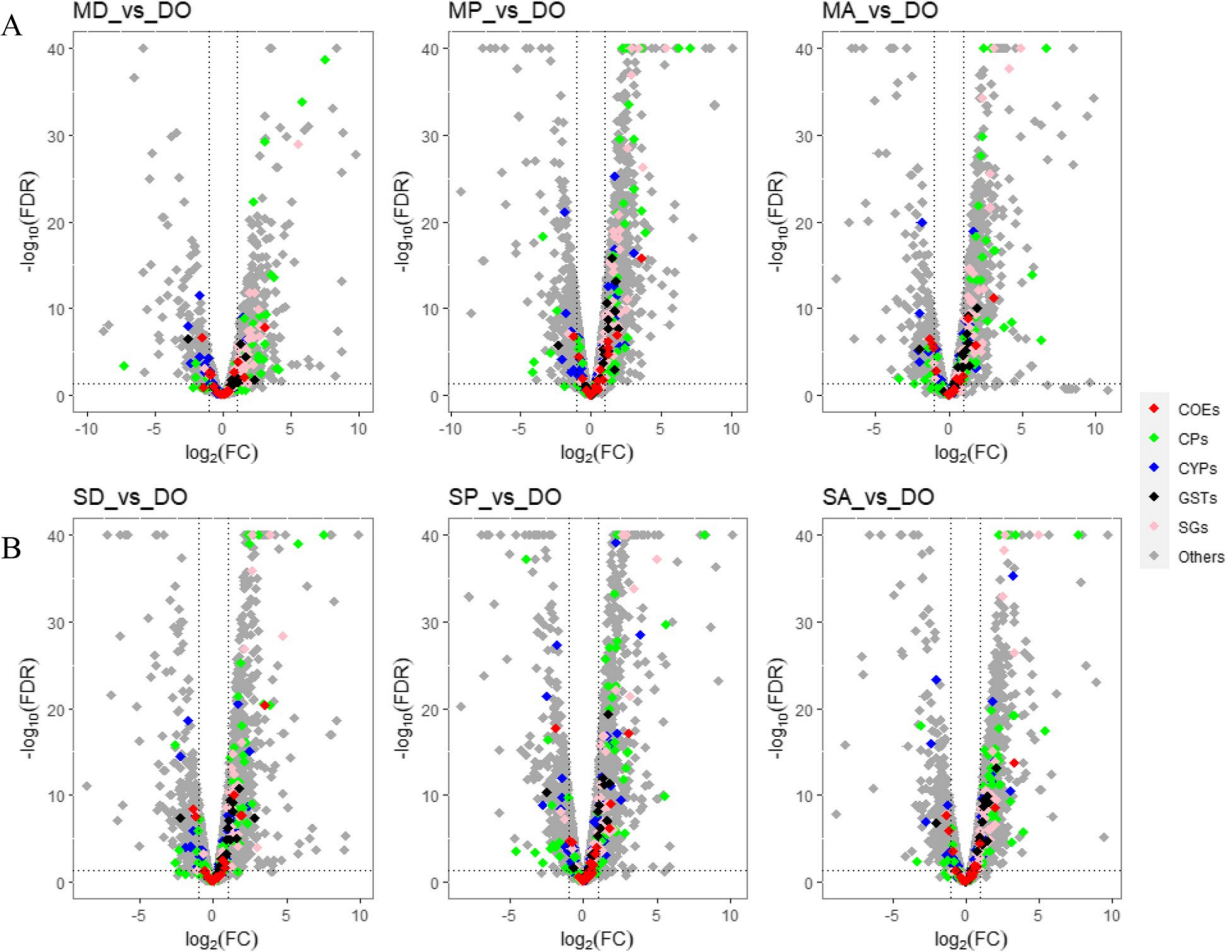
Gene expression profiles of resistant *An. arabiensis* from (**A**) Migori and (**B**) Siaya exposed to deltamethrin, pirimiphos-methyl and alpha-cypermethrin in comparison to the susceptible *An. arabiensis* Dongola strain. The horizontal dotted line on the volcano plot denotes a *P*-value of 0.01 while the vertical dotted lines indicate twofold expression differences. On the x-axis of each plot, genes that are overexpressed in the population are > 0. The -log10FDR values greater than 40 are displayed as 40. Generally, differentially expressed genes associated with cuticular proteins (CPs) and salivary gland proteins (SGPs) were over-expressed at higher proportions in all the six comparisons. COE = carboxylesterases; CP = cuticular protein; CYP = cytochrome P450s monooxygenases; GST = glutathione S-transferases; SGP = salivary gland protein

Consistent with the alphacypermethrin results, in all Res-Sus DELTA comparisons from both sites, most of DEGs associated with CPs and SGPs were overexpressed at higher proportions (Fig. 3). In Migori, 89% CPs (34/38), 96% SGPs (27/28), and 67% GSTs (2/3) DEGs were up regulated in the Res-Sus comparisons, respectively. In Siaya, 89% CPs (33/37), 96% SGPs (22/23), and 89% GSTs (8/9) were up regulated, respectively (Additional file 6).

Identification of core genes associated with DELTA resistance was done by selecting genes commonly shared in the Res–Sus comparisons from both sites (Fig. 2B, Additional file 7). A total of 32 (30 up and 2 down regulated) cuticular proteins and 19 up regulated salivary gland proteins were associated with DELTA resistance at both sites. Some of the cuticular protein genes included: CPAP3-A1b, CPAP3-A1c, CPR10, CPR15, CPR16 and CPR9 while some of the salivary gland protein genes included D7L1 and D7L2. There was a total of 14 detoxification genes which were significantly differentially expressed in the DELTA Res-Sus comparisons. These included the following 8 up regulated genes: CYP307A1, CYP4C27, CYP9L1, CYP9M1, GSTE4 & 3 COEs as well as the following 6 down regulated genes: CYP325D1, CYP325H1, CYP4C35, CYP4J10, GSTE1 and a COE (Table 2).

### Differential gene expression associated with pirimiphos-methyl resistance.

In Migori, a pairwise comparison between pirimiphos-methyl resistant and susceptible mosquitoes (MP vs DO) had a total of 1507 differentially expressed genes (1007 up and 500 down regulated). A pairwise comparison of pirimiphos-methyl resistant and unexposed mosquitoes (MP vs MU) had a total of 357 differentially expressed genes (193 up and 164 down regulated) while a pairwise comparison of unexposed to susceptible (MU vs DO) mosquitoes had a total of 1101 differentially expressed genes (630 up and 471 down regulated) (Table 1, Additional file 4A). In Siaya, a total of 1496 genes (987 up and 509 down regulated) were significantly differentially expressed in pirimiphos-methyl resistant as compared to susceptible mosquitoes (SP vs DO). A comparison between pirimiphos-methyl resistant and unexposed mosquitoes (SP vs SU) had a total of 68 differentially expressed genes (42 up and 26 down regulated) while a comparison of unexposed and susceptible mosquitoes (SU vs DO) had a total of 1155 differentially expressed genes (815 up and 340 down regulated) (Table 1, Additional file 4B).

Focusing on the significantly differentially expressed genes overlapping the Res-Sus and Res-Con comparisons, Migori had a total of 116 genes (94 up and 22 down regulated) (Additional file 4A). Among the top genes which were up regulated and had retrievable annotations, there were CYP450s (CYP6M2, CYP6P2, CYP6Z3), 2 cuticular proteins, a probable chitinase 10 and a synaptic vesicle glyco 2B-like. Siaya had a total of 41 genes (29 up and 12 down regulated) that overlapped the Res-Sus and Res-Con comparisons (Additional file 4B). The top 15 up regulated genes included a CYP450 (CYP9M1), a multidrug resistance-associated 1-like gene, a chitinase partial and a serine protease. Five DEGs were found to overlap all three pairwise comparisons, including CYP450s (CYP6M2 and CYP6Z3) and a carboxylesterase (AARA007309) (Additional file 5). The consistent overexpression of the CYP6M2 and CYP6Z3 in the primiphos-methyl resistant mosquitoes from both Siaya and Migori independent of which group they were compared to (Con or Sus), highlights their potential contribution to the detoxification of this insecticide.

Similar to the ACYP and DELTA results, the gene expression profiles of PMM resistant *An. arabiensis* from both Migori and Siaya showed a higher proportion of CPs and SGPs that were overexpressed in the Res-Sus comparisons (Fig. 3). In Migori, 88% (37/42), 97% (32/33), and 91% (10/11) of the DEGs associated with CPs, SGPs, and GSTs were up regulated in the Res-Sus comparisons, respectively. In Siaya, 74% CPs (32/43), 93% SGPs (27/29), and 90% GSTs (9/10) were up regulated, respectively, in the Res-Sus comparisons (Fig. 3, Additional file 6).

Identification of core genes associated with PMM resistance was done by selecting genes commonly shared in the Res–Sus comparisons from both sites (Fig. 2C, Additional file 7). A total of 32 (27 up and 5 down regulated) CP and 26 (25 up and 1 down regulated) SGP genes were associated with PMM resistance (Fig. 2C, Additional file 7). Some of the up regulated genes included CPAP3-A1b, CPR10, CPR15, CPR16 & CPR9 cuticular proteins as well as D7L1 and D7L2 salivary gland proteins. Among the detoxification genes present, 21 (11 CYP450s, 7 GSTs and 3 COEs) were up regulated while 7 (5 CYP450s, 1 GST and 1 COE) were down regulated. Some of the up regulated genes included CYP4C27, CYP6M2, CYP6M3, CYP9L1, CYP9M1, GSTE4 and 3 COEs. The down regulated genes included CYP306A1, CYP325D1, CYP325H1, CYP4C35, CYP9M2 GSTE1 and 1 COE (Table 2).

### Shared genes associated with ACYP, DELTA and PMM insecticide resistance across collection sites

**Fig. 4:**
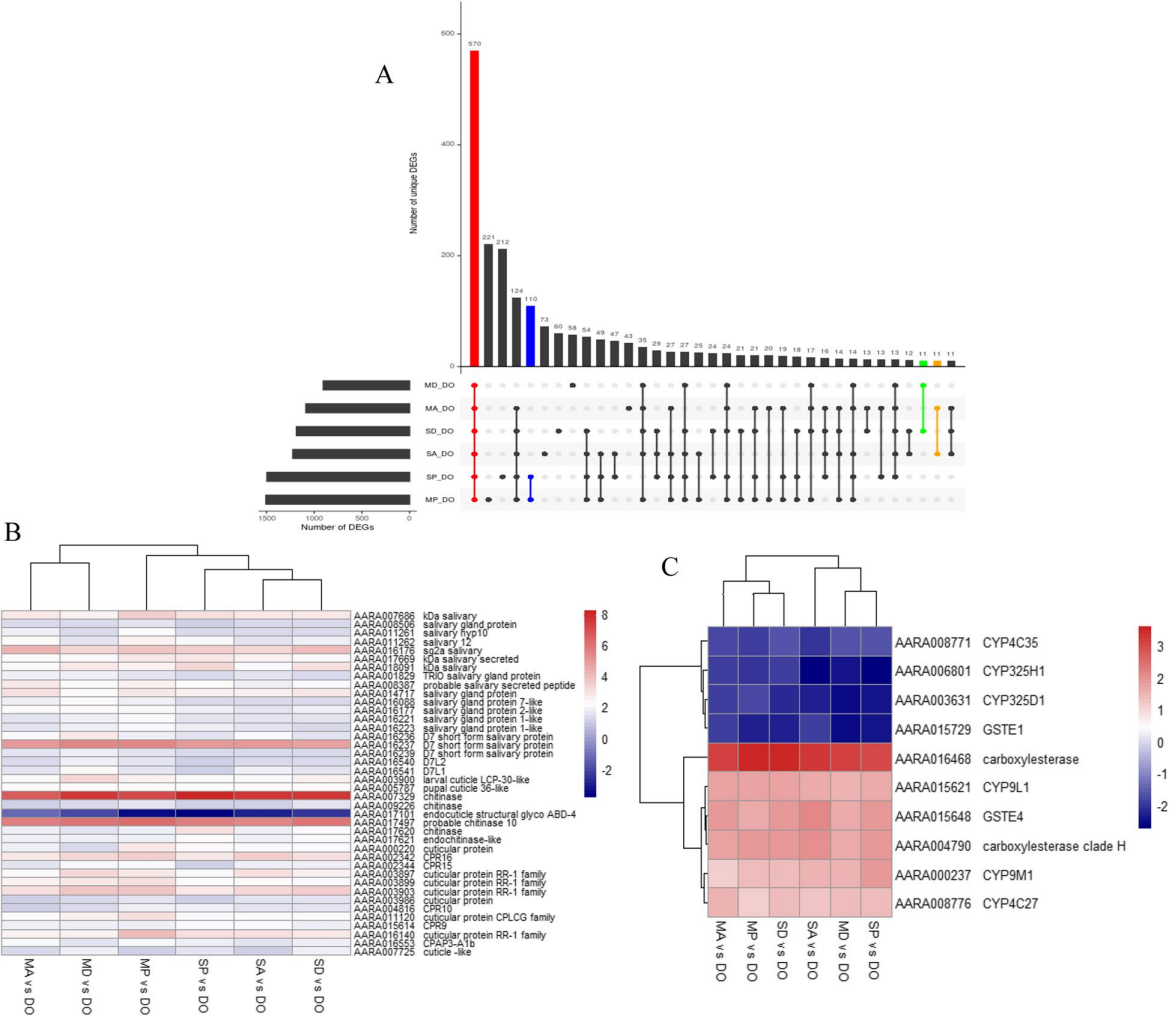
Identification of DEGs associated with resistance to multiple insecticides. **A** Upset plot representing the intersection of DEGs between DELTA, ACYP, PMM resistant mosquitoes from Siaya and Migori when compared to the susceptible *An. arabiensis* Dongola strain (Res-Sus comparisons). The left horizontal bar plot (set size) reports the total number of DEGs in each comparison, the circles represent the set of comparisons associated with the intersection, while the vertical bar plot reports the number of unique and overlapped DEGs (intersection size) between the different combinations of the R-S comparisons. The highlighted bar plot represents core DEGs commonly shared across PMM, DELTA and ACYP, (red), DEGs specific to PMM (blue), DELTA (green) and ACYP (yellow). **B** Heatmap of the log2 fold change (log2FC) expression of all cuticular and salivary gland protein core DEGs that were differentially expressed in all the resistant vs susceptible pairwise comparisons from both sites. **C** Heatmap representing the log2FC expression of the top 10 detoxification genes shared between all insecticide resistant vs susceptible pairwise comparisons for both sites. The heatmaps are in a blue-red color gradient (red = over-expressed and blue = under-expressed). SA = Siaya alpha-cypermethrin, SD = Siaya deltamethrin, SP = Siaya pirimiphos-methyl, SU = Siaya unexposed, MA = Migori alpha-cypermethrin, MD = Migori deltamethrin, MP = Migori pirimiphos-methyl, MU = Migori unexposed, DO = Dongola (*An. arabiensis* susceptible strain)

From both Siaya and Migori, comparing across ACYP, DELTA and PMM resistant and susceptible mosquitoes, 570 genes (446 up and 124 down regulated) were significantly differentially expressed (Fig. 4A). The following up regulated genes were among the top 20 which had retrievable annotations: 2 chitinases, 2 serine protease 53-like, a salivary gland protein, 2 thioester-containing partial and a neuronal acetylcholine receptor subunit.

Among the 570 genes, there were 21 (20 up and 1 down regulated) cuticular proteins, 19 up regulated salivary gland proteins (Fig. 4B), 6 (3 up and 3 down regulated) CYP450s, 2 (1 up and 1 down regulated) GSTs and 2 up regulated COEs which were shared across sites and insecticides (Fig. 4C). Some genes of interest included a glutactin-like COE (AARA016468; FCs = 8.53, 8.68, 8.90, 10.13, 11.74 and 12.09), and cuticular protein genes such as a cuticle-like CPR16 (AARA002342; FCs = 6.41, 7.41, 8.73, 9.27, 10.46 and 11.82), an endocuticle structural glyco ABD-4 (AARA003903; FCs = 6.91, 8.73, 9.70, 11.19, 12.53 and 13.39) and an endocuticle structural glyco ABD-5-like (AARA016140; FCs = 6.08, 6.09, 6.55, 7.68, 8.28 and 15.14). Focusing on insecticide-specific genes, a total of 110 genes were associated only with PMM resistance while 11 genes were associated with DELTA and ACYP resistance (Fig. 4A). Genes such as CYP325K1 and AARA017332 whose ortholog in *An. gambiae* is COEJHE2E were specific to PMM resistance in addition to cuticular (AARA005785 and AARA007248) and salivary gland protein genes (AARA016215). The CYP4J10 gene was only specific to DELTA resistance. No gene with functional validation associated with insecticide resistance was present for ACYP.

### Gene Ontology enrichment analysis

**Fig. 5:**
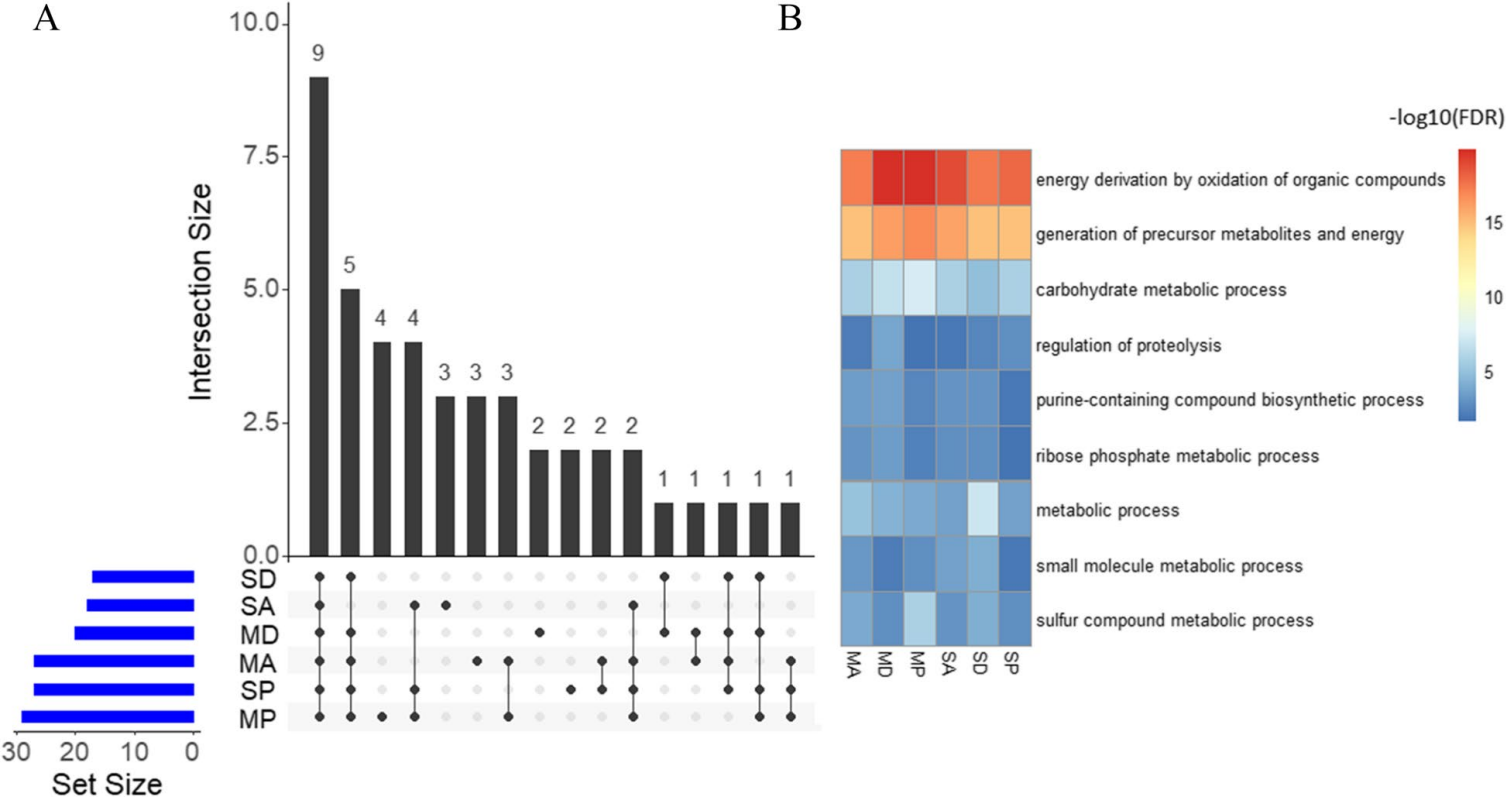
Gene ontology enrichment analysis of the overexpressed genes. The upset plot (**A**) depicts the number of unique and shared GO terms from the functional enrichment analysis. The horizontal bars (blue) indicate the number of enriched GO terms in each comparison (Res vs Sus), while the vertical bars (black) represent number of overlapping GO terms in the sets, indicated with black dot under each bar. The heatmap (**B**) represents the -log10(FDR) of the 9 enriched GO terms that overlap all the comparisons. GO enrichment analysis results are shown for only the overexpressed genes detected in the resistant group vs susceptible (Res vs Sus). The complete report of GO enrichment analysis is shown in Additional file 8

Gene Ontology (GO) enrichment analysis of the DEGs detected from each Res-Sus comparison (n = 6) was conducted using Goatools [19]. The list of enriched GO terms associated with the up and down regulated genes for each comparison is reported in Additional file 8. The overlap of the significantly enriched GO Biological Process (BP) terms across the 6 comparisons is depicted Fig. 5A, while the enriched GO terms of the overexpressed genes that overlapped all the six comparisons are shown in Fig. 5B. A total of nine GO terms were significantly enriched in all the comparisons, including “carbohydrate metabolic process” (GO:0005975), “energy derivation by oxidation of organic compounds” (GO:0015980), “generation of precursor metabolites and energy” (GO:0006091), “metabolic process” (GO:0008152), “purine-containing compound biosynthetic process” (GO:0072522), “regulation of proteolysis” (GO:0030162), “ribose phosphate metabolic process” (GO:0019693), “small molecule metabolic process” (GO:0044281), and “sulfur compound metabolic process” (GO:0006790). Not surprisingly, the number of enriched GO terms was positively correlated with the number of DEGs for the six comparisons, suggesting that the change in metabolic pathway level in mosquitoes is strongly associated with the changes in the expression of individual genes.

### Genetic variation analyses on Voltage-Gated Sodium Channel (Vgsc) and acetylcholinesterase (Ace-1) genes

RNA-Seq reads from pools of 10 mosquitoes in each representing the ACYP, DELTA and PMM experimental replicates were used for Single Nucleotide Polymorphism (SNP) analysis to approximate the allele frequencies at target site mutations in the kdr, ACE-1 and GSTE2 loci. With a focus on the non-synonymous SNPs on the Vgsc gene described by Clarkson*, *et al*.* [20], L995S was detected at a frequency of 33% in Migori and 17% in Siaya ACYP resistant mosquitoes and L995F was detected at 50% in both Siaya ACYP and DELTA resistant mosquitoes. No SNPs were detected in the ACE-1 and GSTE2 genes (Additional file 9). These results suggest that insecticide resistance in the study populations is mainly of a metabolic nature.

### Validation of gene expression levels estimated by RNA-Seq using Quantitative Real-Time PCR (qRT-PCR)

**Fig. 6:**
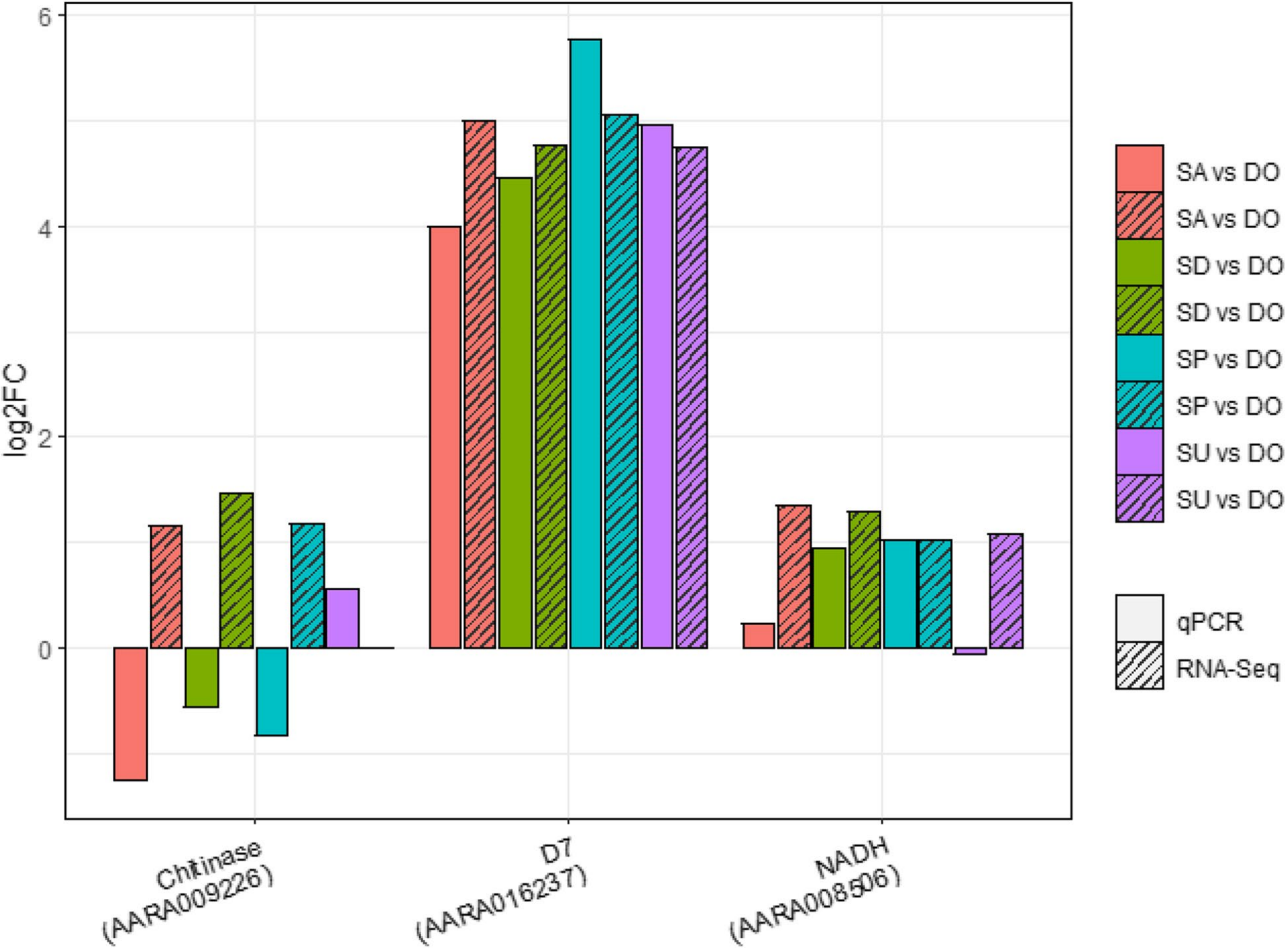
qPCR validation of RNA-Seq gene expression levels in Siaya alpha-cypermethrin (SA), deltamethrin (SD), pirimiphos-methyl (SP) and unexposed (SU) mosquitoes, versus the susceptible Dongola strain (DO)

To complement the RNA-Seq results, we conducted qRT-PCR to validate some of the differentially expressed genes including chitinase, salivary gland protein, NADH dehydrogenase and the D7 genes alongside two housekeeping genes, RPS7 and Actin5c. Most of the qRT-PCR data including the D7 and NADH endorsed the directionality of expression as estimated by RNA-Seq Fig. 6. However, qRT-PCR of the chitinase gene was not congruent with the RNA-Seq results, likely due to the low transcriptional signal of this gene as reflected in the RNA-Seq (Additional file 10).

## Discussion

High throughput sequencing platforms have enabled genomic and transcriptomic-level studies of the genetic profiles of insecticide resistant malaria vectors. The overarching goal of this study was to investigate the transcriptomic profiles of DELTA, PMM and ACYP resistant *An. arabiensis* from western Kenya. Results from this study showed that *An. arabiensis* from the counties of Siaya and Migori had varied levels of phenotypic resistance to DELTA, PMM and ACYP. This resistance was associated with elevated expression of salivary gland and cuticular protein genes in addition to detoxification genes including CYP450s, COEs and GSTE.

The phenotypic resistance in the *An. arabiensis* populations from Siaya and Migori varied across the different insecticides. The higher frequency of PMM resistance compared to the two pyrethroids was unexpected. The use of insecticides in agriculture could contribute to selective pressure since some of the insecticides used in agriculture contain the same active ingredients as those used for vector control [21]. Whereas pyrethroid resistance had been previously reported in *An. arabiensis* from western Kenya at varying intensities, the vectors remained susceptible to organophosphate insecticides [12, 22]. The emergence of resistance to organophosphates may be associated with run off from insecticides used in agriculture into larval habitats [3, 23]. In addition, the use of organophosphates has now been introduced for IRS in western Kenya and continued usage will likely result in increasing resistance over time.

In both sites, there was a high level of overexpression of cuticular and salivary gland proteins, as well as detoxification genes, some of which have been previously reported [17, 18]. Salivary gland proteins are known to play important roles during blood feeding in mosquitoes such as releasing anti-coagulants and are thus important to transmission of malaria parasites [24]. In other cases, the SGPs aid in transmission of viruses by mosquitoes [25]. Here, we report overexpression of twelve salivary gland proteins present in insecticide resistant mosquitoes which included the D7 long (D7L1 and D7L2) and short form which have previously been associated with insecticide resistance [18]. Interestingly, eleven of these genes have been found to be present in *An. arabiensis* populations from Ethiopia, some of which were found to be overexpressed in the pyrethroid and organophosphate resistant group [17] and may point to their contribution to insecticide resistance in *An. arabiensis* across the East African region.

The ortholog of the D7 short form gene (AARA016237) in *An. gambiae,* D7r4, was found to be overexpressed in carbamate resistant mosquitoes [18]. The presence of D7r4 gene in both *An. arabiensis* and *An*. *gambiae* resistant to pyrethroid, organophosphate and carbamate insecticides could suggest the possibility of a cross resistance mechanism [17, 24]. A recent study by Freitas and Nery [24] identified several SGPs which could be considered as potential targets for novel vector control strategies. Further investigation will be necessary to ascertain the role of SGP genes associated with insecticide resistance.

Cuticular protein genes including CPAP3-A1b, CPR9, CPR10, CPR15 and CPR16 were significantly differentially expressed in mosquitoes showing resistance to all the three insecticides from both sites. The insect cuticle is mainly composed of chitin and cuticular proteins such as those of the CPR and CPAP families which protects the insect from harsh weather conditions. Any alterations to the cuticle composition could lead to changes such as thickening which may impede insecticide penetration and render the mosquitoes resistant. Some cuticular proteins such as the CPAP3, CPR and CPLCG families observed in the resistant mosquitoes in this study have previously been associated with resistance to different insecticides in *Anopheles* mosquitoes [26–29]. A study done by Zoh, et al. [29] showed that the CPAP3-A1b gene was linked to clothianidin (neonicotinoid) resistance in *An. gambiae s.s* from Côte d'Ivoire (Tiassale)*.* This gene was also overexpressed in pyrethroid and organophosphate resistant *An. arabiensis* samples analyzed in this study and also in pyrethroid resistant *An. arabiensis* from Tanzania [30]. Although this gene can be considered as a potential marker for insecticide resistance, a further functional verification step is needed to confirm the role of the CPAP3-A1b gene in both *An. gambiae* and *arabiensis* populations resistant to different classes of insecticides. Additional studies by Yahouédo, et al. [28] Zhou*, *et al*.* [31] have provided evidence on the presence of genes belonging to the CPR and CPLCG families in insecticide resistant *Anopheles* mosquitoes.

Some members of the CYP450s, such as the CYP4G family, are involved in cuticle development by catalyzing the production of cuticular hydrocarbons, the most abundant lipid species in the epicuticle [32]. Studies conducted by Balabanidou, et al. [32] and Yahouédo, et al. [28] demonstrated that insecticide resistant mosquitoes had thicker cuticles compared to susceptible mosquitoes due to the overexpression of the cuticular protein genes that led to enriched deposition of CHCs. It will be important to further investigate the role of CYP540 genes overexpressed in resistant samples to determine their contribution to cuticle development.

In both Migori and Siaya, several detoxification genes were overexpressed in mosquitoes showing resistance to all insecticides. These detoxification genes included CYP9M1, CYP4C27, CYP9L1, GSTE4 and two COEs (AARA004790 and AARA016468). Previous studies have reported the presence of these genes, with the exception of CYP9M1, in mosquitoes resistant to insecticides including pyrethroids and organophosphates [17, 33–35]. Their presence in our study further supports their association with resistance and their potential for use as molecular insecticide resistance markers. Genes such as GSTE4, CYP9L1 and the two COEs (AARA004790 and AARA016468) have previously been identified in resistant *An. arabiensis* and might be considered as species-specific markers for resistance to pyrethroids and organophosphates [17]. GSTs belonging to the delta and epsilon classes have previously been demonstrated to metabolize insecticides including pyrethroids, organophosphates and organochlorines [33, 36–41]. Here, GSTE4 was found to be over-expressed across all samples from both sites resistant to pyrethroids and organophosphate. Although the role of GSTE4 in metabolizing insecticides is not fully understood, a previous study [42] suggests that this enzyme could be involved in sequestration of insecticides. Functional validation will be required to understand the role of GSTEs including GSTE4 in insecticide resistance in *An. arabiensis*.

Besides genes that were associated with multiple insecticide resistance, some were specific to the different insecticides. Focusing on genes with functional validation associated with insecticide resistance, CYP4J10 was found to be specific to only DELTA. This gene has previously been associated with resistance to permethrin which is also a pyrethroid [43]. Genes such as CYP325K1 which was specific to PMM resistance has been associated with pyrethroid resistance in *An albimanus* and *Aedes aegypti* [16, 44]. The gene AARA017332 whose ortholog in *An. gambiae* is COEJHE2E has been linked with permethrin resistance in *An. arabiensis* and here, PMM resistance suggesting that it might not be an insecticide specific gene [33]. In addition to the detoxification genes, there were some cuticular (AARA005785 and AARA007248) and salivary gland protein genes (AARA016215) which were specific to PMM but have not previously been associated with resistance.

Taken together these results describe the suite of marker genes involved in *An. arabiensis* resistance to key pyrethroid and organophosphate insecticides used in malaria vector control. Once validated, they can be considered as candidate molecular markers that National Malaria Programs can use to routinely monitor for the emergence of insecticide resistance. In addition, these results add to the growing body of knowledge on the molecular basis of insecticide resistance within key vector populations [45].

## Conclusion

In this study, *An. arabiensis* populations from Siaya and Migori counties were found to be resistant to ACYP, DELTA and PMM insecticides at different levels. Furthermore, transcriptomic analysis revealed novel resistance genes in Kenyan *An. arabiensis* in addition to those that have previously been described in other countries. Gene expression profiles showed an overexpression of the salivary gland proteins belonging to both the short and long form D7 genes, and cuticular proteins (including CPR9, CPR10, CPR15, CPR16) that were shared between pyrethroid and organophosphate resistant mosquitoes. In addition, detoxification genes including CYP450s (CYP9M1, CYP325H1, CYP4C27, CYP9L1 and CYP307A1), 2 COEs and a GSTE (GSTE4) were also over-expressed. A functional validation of these markers is needed to confirm the role these markers in conferring insecticide resistance.

## Methods

### Larval sampling and rearing

**Fig. 7:**
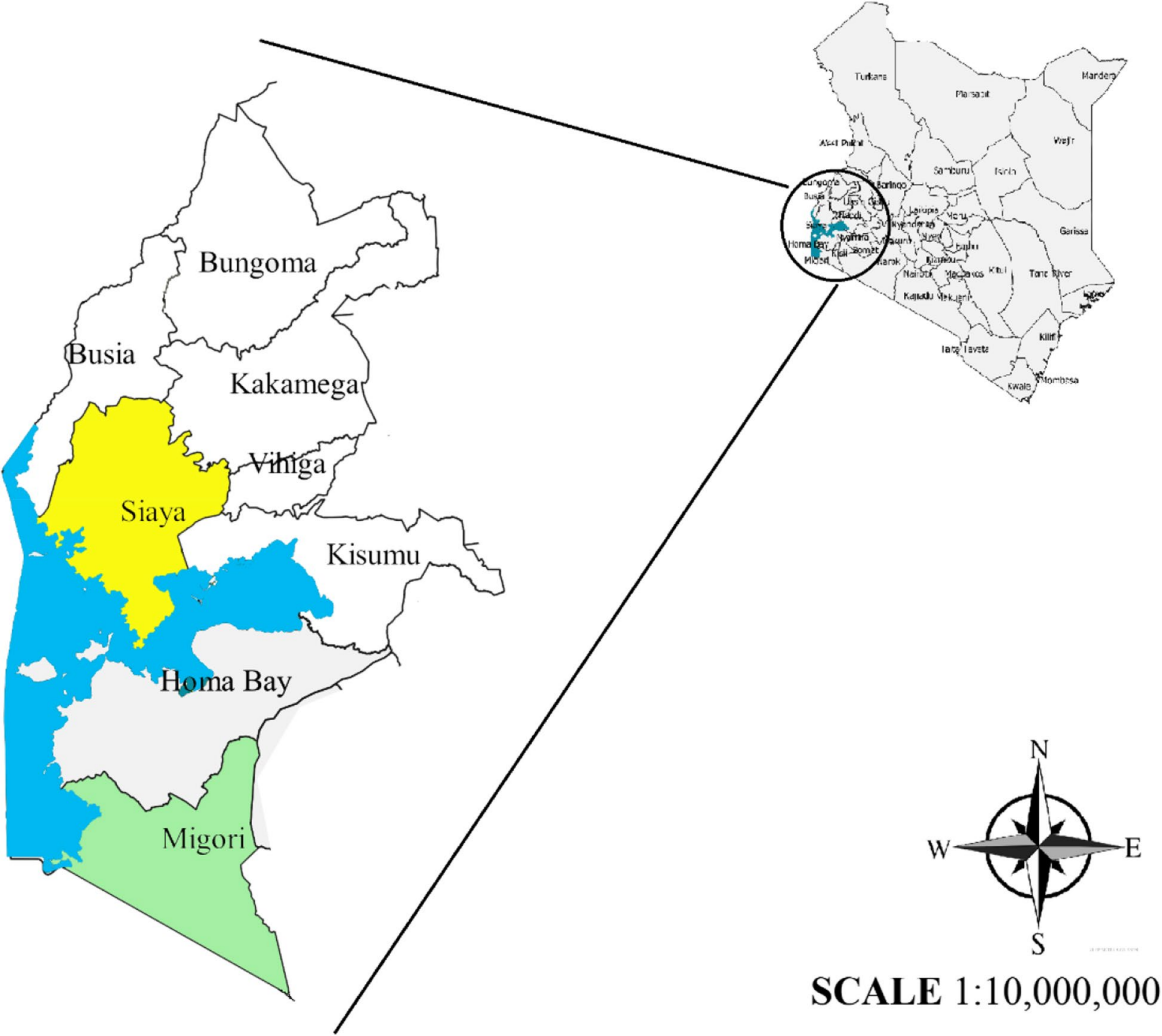
Map of Kenya (right) showing Migori and Siaya counties in expanded view where *An. arabiensis* were collected

Sampling of *Anopheles* larvae in Migori (1.0707° S, 34.4753° E) and Siaya (0.0626° N, 34.2878° E) Fig. 7 was conducted between August and October 2019. Larvae were sampled using dippers and placed in collection tins with labels indicating the collection date and site. The collected larvae were transported to and reared using standard methods described by Benedict [46] at the insectary at the Centre for Global Health Research, Kenya Medical Research Institute, Kisumu (KEMRI- CGHR). Resulting adult mosquitoes were sustained on a 10% sugar solution soaked in cotton balls and held for 3–5 days before exposure to insecticides for susceptibility testing. All the collected larvae from both sites were reared under temperatures of (30 ± 2 °C), while adults were reared at a temperature of 27 ± 2 °C, relative humidity of 80 ± 10%, and photoperiod of 12:12 light: dark cycle.

### Insecticide resistance phenotyping

CDC bottle bioassays were conducted on the 3–5 days old F_0_ adult female mosquitoes following the U.S. Centers for Disease Control and Prevention (CDC) guidelines for evaluating insecticide resistance [47]. Mosquitoes from both Migori and Siaya were exposed to ACYP, DELTA and PMM insecticides at varying concentrations ranging from 0.5X to 5X the diagnostic doses. Technical grade insecticides were diluted using acetone in 50 ml falcon tubes to obtain stock diagnostic concentrations of 12.5 μg/bottle for ACYP, 12.5 μg/bottle for DELTA and 20 μg/bottle for PMM (Additional file 11).

Each experiment comprised of 1 control bottle treated with 1 ml of acetone and 4 test bottles each treated with 1 ml of the respective insecticide solution and left overnight to dry except for the PMM bottles which were dried for only 3 h prior to bioassay. Approximately 15–20 mosquitoes were introduced in each bottle using a mouth aspirator and exposed for the diagnostic time of 30 min. Mosquitoes were considered resistant if they were capable of standing and flying in a coordinated manner and susceptible if they died or were moribund. The unexposed mosquitoes were the field mosquitoes used in the control bottle that contained no insecticide. Resistant and unexposed mosquitoes were knocked down on ice to immobilize them then 2 -3 legs were cut and placed in a sterile 1.5 ml Eppendorf tube for species identification. The remaining carcass was preserved individually in a 1.5 ml Eppendorf tube containing RNA later and stored at 4 °C until shipment for sequencing at the U.S. Centers for Disease Control and Prevention (CDC) in Atlanta, USA.

### Molecular species identification

Genomic DNA was isolated from the legs of the mosquitoes using the ethanol precipitation procedure [48]. 1μl of DNA from each sample was utilized as a template for the PCR process, along with known *An. arabiensis* DNA as a positive control [49]. The reactions were carried out using a BIORAD T100 thermal cycler under the following conditions: 95°C for 5 min, 30 cycles of: 95°C for 30 s, 56°C for 30 s, and 72°C for 30 s, and a final extension at 72°C for 5 min. Primers used were specific to *An. arabiensis*—5'AAG TGT CCT TCT CCA TCC TA 3', *An. gambiae* -5' CTG GTT TGG TCG GCA CGT TT 3' and a universal primer—5' GTG TGC CCC TTC CTC GAT GT 3'. A 2% agarose gel stained with ethidium bromide was used to visualize the 315bp amplicons for *An. arabiensis.*

### RNA extraction, RNA-Seq library preparation and sequencing

RNA-Seq analysis included 3 groups of mosquitoes: susceptible *An. arabiensis* from the Dongola reference strain, resistant, and unexposed *An. arabiensis* from Migori and Siaya counties. The samples were labelled as DO (susceptible *An. arabiensis* Dongola strain), MA (Migori ACYP resistant), MP (Migori PMM resistant), MD (Migori DELTA resistant), MU (Migori unexposed), SA (Siaya ACYP resistant), SD (Siaya DELTA resistant), SP (Siaya PMM resistant), SU (Siaya unexposed). Total RNA isolation was carried out for 3 replicates of each group, each of which contained a pool of 10 mosquitoes. This was done using the Arcturus® PicoPure® RNA isolation kit (Life Technologies, USA) and quantification was carried out using the 4200 TapeStation System (Agilent Technologies, Palo Alto, CA, USA), according to the manufacturers’ protocols. RNA was treated using Baseline-ZERO™ DNase (Epicentre, Illumina) and removal of ribosomal RNA was done using the Ribo-Zero rRNA removal kit (Human/Mouse/Rat) (Epicentre, Illumina). The ScriptSeq v2 RNA-Seq Library Preparation Kit (Epicentre, Illumina) was used to prepare the individual libraries according to the manufacturer's instructions. Equimolar amounts of each library were pooled and sequenced (2 × 125 bp paired—end) on an Illumina HiSeq 2500 sequencer, using v2 chemistry. Sequencing was carried out at the Biotechnology Core Facility at CDC in Atlanta, USA.

### RNA-Seq data analysis

#### Quality control filtering and mapping

For each sample, FastQC v0.11.5 was used to assess the quality of de-multiplexed paired end reads generated after sequencing [50]. Sequencing reads were trimmed and filtered using fastp v0.20.1 [51] to remove adapter and low-quality reads. Parameters used in fastp included a minimum base quality value of 20, required minimum length of 50, trimming of polyG tail and trimming of polyX in the 3’ ends to remove the low-complexity consecutive bases. Subsequently, the raw sequencing reads from similar sequencing lanes of R1 (forward) and R2 (reverse) were concatenated to increase the read depth to be used in subsequent downstream analysis. Trimmed reads were then aligned to the *An. arabiensis* Dongola reference genome assembly (genome assembly version: AaraD1, GeneBank assembly identifier = GCA_000349185.1) downloaded from VectorBase (release 48) using ‘subjunc’ v2.0.1, part of the subread aligner package with default parameters [52].

The resulting alignment was filtered to remove reads of low mapping quality (q < 10) and sorted using Samtools v1.10 [53]. Tag counting was done using ‘*featureCounts*’, part of the subread aligner package. Tags were defined as either a read pair or single, unpaired read. Aligned reads with at least 1 bp overlap in coding sequence (CDS) features in the sense orientation were counted, and the tabulated tag counts used as input for differential expression analysis with edgeR [54].

#### Differential gene expression analysis

Differential gene expression analysis was conducted for all groups. Prior to the analysis, genes that had a total tag count of more than 30 across all libraries were considered while the rest were filtered out to remove the lowly expressed genes. Variation in RNA-Seq data was modelled using a negative binomial distribution and a generalized linear model. The estimated log2 fold change (FC) for each gene was tested in edgeR using a Likelihood-Ratios (LR) test. P-values associated with log_2_FC were adjusted for multiple testing using the False Discovery Rate (FDR) approach and significance was defined as genes differentially expressed with an FDR-adjusted *P*-value < 0.01 [55].

In each site and for each insecticide, three pairwise comparisons were made between resistant *vs* susceptible (Res-Sus), unexposed *vs* susceptible (Con-Sus) and resistant *vs* unexposed (Res-Con). The unexposed mosquitoes are hereafter referred to as the control (Con) group. Res-Sus comparisons were to account for genes differentially expressed because of constitutive differences related to resistant phenotypes. Res-Con comparisons were to identify the genes which could have been induced because of exposure to the insecticides. Con-Sus comparisons were to explain overexpressed genes which are always present within a population since they are transcribed constitutively. Genes which were significantly differentially expressed at false discovery rate (FDR) < 0.01 and fold change (FC) > 2 were considered potential candidate markers for resistance. Of particular interest were the genes which were consistently and significantly differentially expressed across the different pairwise comparisons as these could potentially have been involved in cross resistance.

#### Gene ontology annotation and functional enrichment analysis

The gene ontology and functional annotation of the AaraD1.11 gene set (Genome version = GCA_000349185.1) were performed locally using blast2GO command line v1.4.4 [56] as follows. First, a local BLASTp (v2.9) search of the predicted protein coding sequences was conducted against the Arthropoda (taxid = 6665) category of the nr protein NCBI database with maximum e-value cut-off 10–3. Second, the protein sequences were searched against the InterPro database [57], using InterProScan v5 [58]. The Blastp and InterProScan outputs were simultaneously provided to Blast2GO command line as input, which map the RefSeq and InteProScan identifiers to the GO database as curated and updated in the Blast2GO database (August, 2022).

Subsequently, gene ontology enrichment (GOE) analysis was carried out on differentially expressed gene sets using GOATools [19]. The resulting annotated genes and their associated GO terms were used as reference for this GOE analysis. Fisher’s exact test was used to identify gene ontologies significantly enriched from the over- and under-expressed gene sets relative to the rest of the genome. An FDR adjusted *P-value* < 0.05 was used to determine the significantly enriched GO terms associated with the list of DEGs, while the redundant GO terms were eliminated using REVIGO (available at http://revigo.irb.hr).

#### Genetic variation analyses on Vgsc and Ace-1 genes

The RNA-Seq reads of the insecticide resistant, unexposed, and susceptible groups were mined for non-synonymous SNPs in the Vgsc gene (AARA017729) including V402L, D466H, M490I, G531V, Q697P, T791M, L995S, L995F, V1507I, I1527T, N1570Y, E1597G, K1603T, A1746S, V1853I, I1868T, P1874S, P1874L, A1934V, and I1940T, previously studied by Clarkson, et al. [20] and Messenger, et al. [17]. Additionally, we examined the I114T, L119F, L119V variants in GSTE2 gene (AARA008732) explored by Simma*, *et al*.* [59] and Messenger, et al. [17]. Furthermore, we investigated the G119S in ACE1 gene (AARA001814) previously studied by Weill*, *et al*.* [60], Simma, et al. [59] and Messenger, et al. [17]. The sorted BAM files used for differential gene expression analysis (see above) were used as input files for the SNP analysis using the SAMtools package v1.19 and BCFtools v.1.9 [53]. VCFtools v0.1.17 [61] was then used to generate the allele frequencies of the SNPs, and the variants of interest were extracted.

### Gene validation using qRT-PCR

A quantitative real-time reverse transcription PCR (qRT-PCR) was carried out to validate a set of candidate marker genes that were significantly differentially expressed alongside two *An. gambiae* housekeeping genes: Actin (AGAP000651) and rPS7 (AGAP010592) that also worked with *An. arabiensis* samples. The housekeeping genes were used for normalization of the experiment. RNA was extracted from different mosquitoes of the F_0_ population used for RNA-Seq using the Arcturus™ PicoPure™ RNA Isolation Kit (Applied Biosystems, USA) following the manufacturer’s instructions. The extracted mosquitoes were from three replicates ACYP, DELTA and PMM resistant, unexposed and susceptible Dongola strain mosquitoes. cDNA was synthesized from 1ul of the extracted RNA using the High-Capacity cDNA Reverse Transcription kit (Applied Biosystems, USA) and oligo-d(T)_23_ (New England Biolabs, USA) in accordance with the manufacturer's instructions. A serial dilution of cDNA was used to create standard curves of Ct values for each gene, effectively nullifying any inaccuracies or deviations resulting from sample concentration. The qRT-PCR were performed using the PowerUp SYBR Green Master Mix (Applied Biosystems, USA) on a QuantStudio 3 Real-Time PCR system (Applied Biosystems, USA). Information about the primers including their sequences and their respective gene IDs is as documented in Additional file 12. The thermal cycling conditions were set at 50 ˚C for 2 min, 95 ˚C for 10 min, and thereafter, 40 cycles of: 15 s at 95 ˚C, 1 min at 60 ˚C, followed by 15 s at 95 ˚C, 1 min at 60 ˚C and a final step of 15 s at 95 ˚C. The 2 − ΔΔCT approach was used to calculate the relative expression level and Fold Change (FC) of each target gene from resistant field samples compared to the susceptible lab strain.

### Supplementary Information

**Additional file 1.** Descriptive statistics of RNA-Seq reads and alignments.

**Additional file 2.** Descriptive statistics of feature counts output.

**Additional file 3.** The percentage of tags assigned to features in each replicate of alphacypermethrin, deltamethrin and primiphosmethyl experiments.

**Additional file 4.** Venn diagrams showing the differentially expressed genes in the Res-Sus, Res-Con and Con-Sus pairwise comparisons from both Siaya and Migori.

**Additional file 5.** Functional annotation of the overlapped DEG between Res-Sus, Con-Sus, or Res-Con comparisons for each experiment. All overlapped DEGs belonged to different intersection, for all the six experiments were mapped to their functional description and their fold change expression.

**Additional file 6.** Bar plot comparing the number of up regulated and down regulated DEGs associated with CPs (Cuticular proteins), COEs (Carboxylesterases), CYPs (cytochrome P450s, GSTs (glutathione-S-transferases), and SGs (Salivary gland) proteins.

**Additional file 7.** Functional description and fold change expression of the DEGs that are commonly shared between the insecticide resistant populations of the two sites for each insecticide.

**Additional file 8.** Gene ontology enrichment analysis on the DEGs.

**Additional file 9.** Heatmap showing the allele frequencies of kdr, ACE1 and GSTE2 genes in insecticide resistant samples.

**Additional file 10.** Statistics of differentially expressed genes with annotations from VectorBase and Blast2go.

**Additional file 11.** Concentrations of insecticides used for bioassays.

**Additional file 12.** List of Oligonucleotide primers used for RT-qPCR.

## Data Availability

Sequence data generated by this study is available at Sequence Read Archive (SRA) under the Bio Project accession number PRJNA986474. Custom scripts used for all the analysis are available from the authors on request.

## Abbreviations

ACE1: Acetylcholinesterase
ACYP: Alphacypermethrin
adjP: P-value adjusted for multiple testing
An: Anopheles
BP: Biological process
CDC: Centers for Disease Control and Prevention
COE: Carboxylesterases
Con: Control
CP: Cuticular protein
CYP450s: Cytochrome P450s
DEGs: Differentially expressed genes
DELTA: Deltamethrin
DNMP: Division for National Malaria Program
FC: Fold change
GO: Gene ontology
GSTE: Glutathione-S-transferase
IRM: Insecticide resistance management
IRS: Indoor Residual Spraying
Kdr: Knock down resistance
KEMRI: Kenya Medical Research Institute
LLINs: Long lasting insecticidal nets
NMCP: National Malaria Control Program
PMM: Pirimiphos-methyl
qRT-PCR: Real –time quantitative polymerase chain reaction
Res: Resistant
SG: Salivary gland
SNP: Single nucleotide polymorphism
SSA: Sub-Saharan Africa
Sus: Suceptible
