# Supplementary material for: Whole transcriptomic analysis reveals overexpression of salivary gland and cuticular proteins genes in insecticide-resistant Anopheles arabiensis from Western Kenya
Source: BMC Genomics. 2024 Mar 27;25:313. doi: 10.1186/s12864-024-10182-9 (PMC10967204; doi:10.1186/s12864-024-10182-9)
Supplement: Supplementary file 3 — Additional file 3. The percentage of tags assigned to features in each replicate of alphacypermethrin, deltamethrin and primiphosmethyl experiments. [file 12864_2024_10182_MOESM3_ESM.docx]

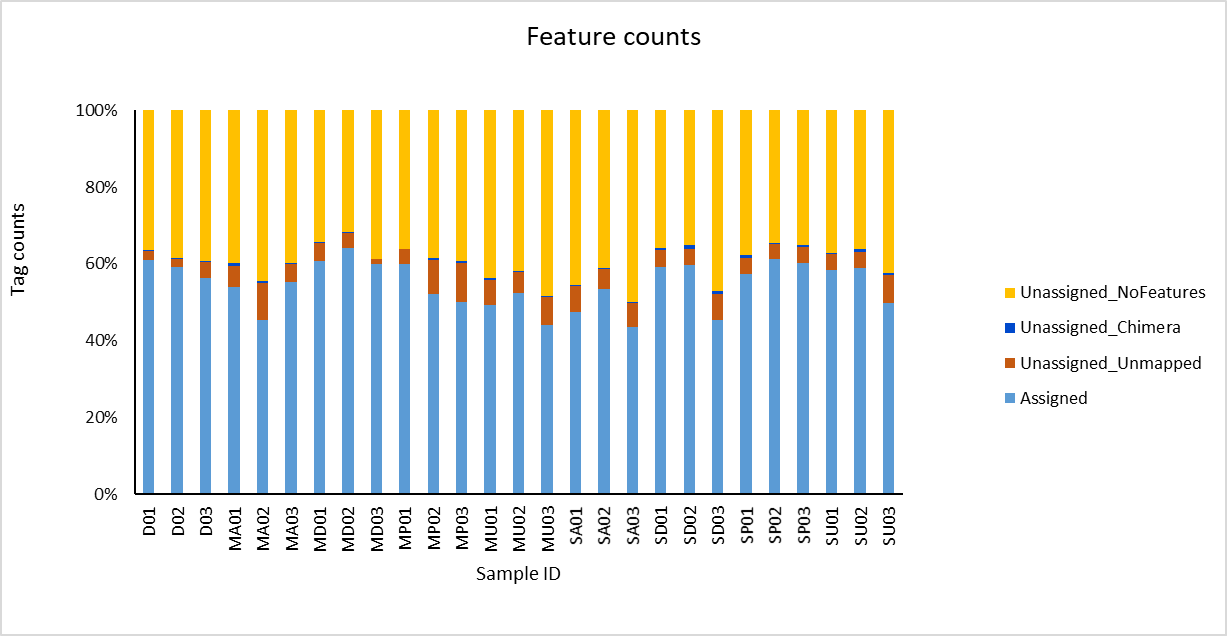


Supplementary Figure 1: summarizing the percentage of tags assigned to features in each replicate of alphacypermethrin, deltamethrin and primiphosmethyl experiments. The percentage of tags assigned to features (in blue) ranged between 48% to 62%. DO= susceptible An. arabiensis Dongola strain, MA = Migori alphacypermethrin, MP= Migori primiphosmethyl, MD= Migori deltamethrin, MU= Migori unexposed, SA= Siaya alphacypermethrin, SD= Siaya deltamethrin, SP= Siaya primiphosmethyl, SU= Siaya unexposed.
