# Supplementary material for: Whole transcriptomic analysis reveals overexpression of salivary gland and cuticular proteins genes in insecticide-resistant Anopheles arabiensis from Western Kenya
Source: BMC Genomics. 2024 Mar 27;25:313. doi: 10.1186/s12864-024-10182-9 (PMC10967204; doi:10.1186/s12864-024-10182-9)
Supplement: Supplementary file 4 — Additional file 4. Venn diagrams showing the differentially expressed genes in the Res-Sus, Res-Con and Con-Sus pairwise comparisons from both Siaya and Migori. [file 12864_2024_10182_MOESM4_ESM.docx]

**Supplementary Figure 2: Differential gene expression analysis**


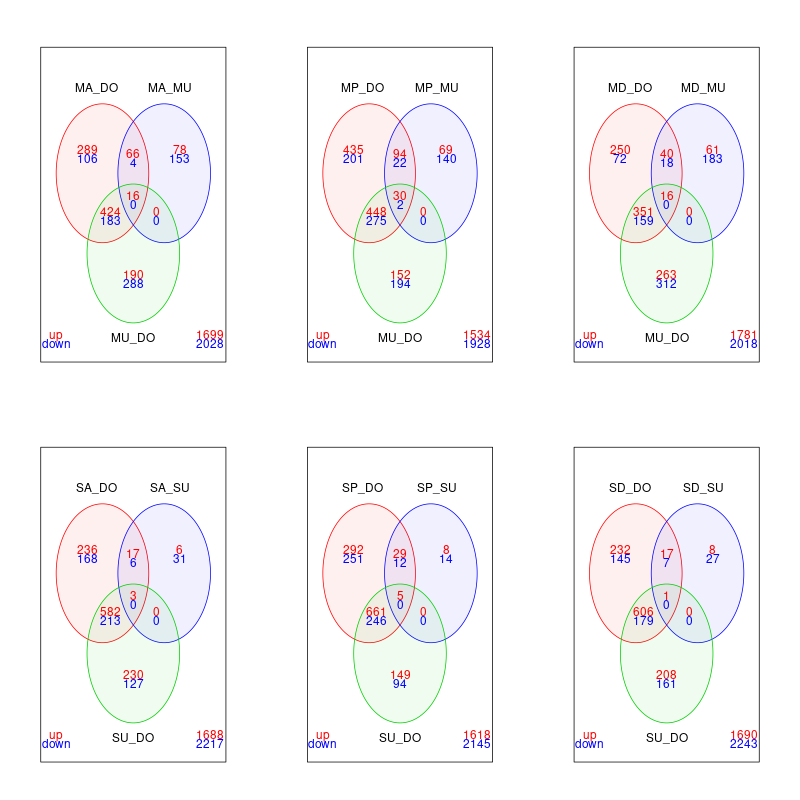


**A**

**B**

Venn diagrams showing the differentially expressed genes in the Res-Sus, Res-Con and Con-Sus pairwise comparisons from both Siaya and Migori (logFC = >=1 or <= -1, FDR = < 0.01). A) Migori B) Siaya alphacypermethrin, primiphosmethyl and deltamethrin experiments, respectively. Up regulated genes are shown in red while down regulated genes are shown in blue.

SA = Siaya alphacypermethrin, SD= Siaya deltamethrin, SP= Siaya primiphosmethyl, SU= Siaya unexposed, MA= Migori alphacypermethrin, MD= Migori deltamethrin, MP= Migori primiphosmethyl, MU= Migori unexposed, DO= Dongola (*An. arabiensis* susceptible strain), DE = differentially expressed, FC = Fold change
