# Supplementary material for: Whole transcriptomic analysis reveals overexpression of salivary gland and cuticular proteins genes in insecticide-resistant Anopheles arabiensis from Western Kenya
Source: BMC Genomics. 2024 Mar 27;25:313. doi: 10.1186/s12864-024-10182-9 (PMC10967204; doi:10.1186/s12864-024-10182-9)
Supplement: Supplementary file 6 — Additional file 6. Bar plot comparing the number of up regulated and down regulated DEGs associated with CPs (Cuticular proteins), COEs (Carboxylesterases), CYPs (cytochrome P450s, GSTs (glutathione-S-transferases), and SGs (Salivary gland) proteins. [file 12864_2024_10182_MOESM6_ESM.docx]

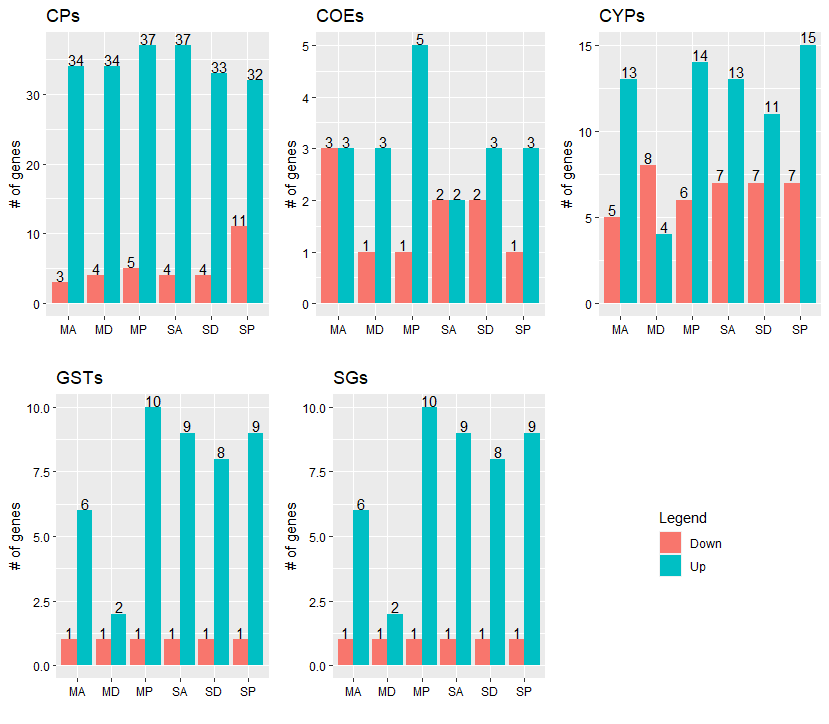


**Supplementary Figure 4**: Bar plot comparing the number of up regulated and down regulated DEGs associated with CPs (Cuticular proteins),COEs (Carboxylesterases), CYPs (cytochrome P450s, GSTs (glutathione-S-transferases), and SGs (Salivary gland) proteins
