# Supplementary material for: Whole transcriptomic analysis reveals overexpression of salivary gland and cuticular proteins genes in insecticide-resistant Anopheles arabiensis from Western Kenya
Source: BMC Genomics. 2024 Mar 27;25:313. doi: 10.1186/s12864-024-10182-9 (PMC10967204; doi:10.1186/s12864-024-10182-9)
Supplement: Supplementary file 9 — Additional file 9. Heatmap showing the allele frequencies of kdr, ACE1 and GSTE2 genes in insecticide resistant samples. [file 12864_2024_10182_MOESM9_ESM.docx]

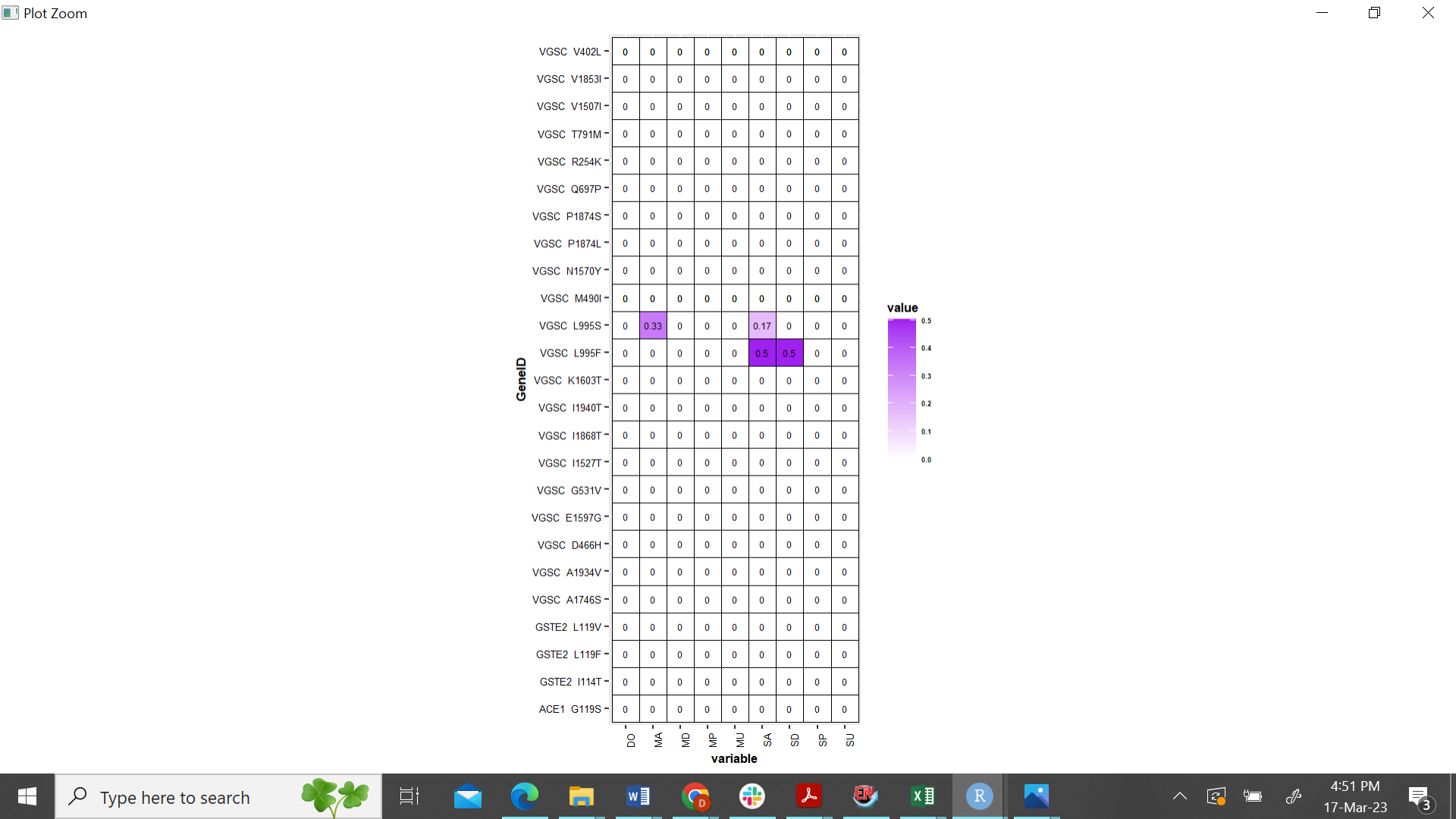


Heatmap showing the allele frequencies of kdr, ACE1 and GSTE2 genes in insecticide resistant samples. Mutant alleles were only present in the kdr gene at positions L995S and L995F.
